# Optimization of Mixture Parameters for Rubber-Modified Permeable Concrete Bricks Using Response Surface Methodology

**DOI:** 10.3390/ma19122660

**Published:** 2026-06-20

**Authors:** Jiaxiong Zhan, Wei Qiao, Yiran Qin, Zhihua Luo, Haoxian Shi, Jing Li

**Affiliations:** 1 School of Engineering and Technology, China University of Geosciences, Beijing 100083, China; 2 State Key Laboratory of Deep Earth Exploration and Imaging, China University of Geosciences, Beijing 100083, China; 3 Guangzhou Marine Geological Survey, Guangzhou 511458, China; 4 National Engineering Research Center for Natural Gas Hydrate Exploration and Development, Guangzhou 511458, China

**Keywords:** permeable concrete brick, waste tire rubber particles, response surface methodology, compressive strength, permeability coefficient

## Abstract

Permeable concrete bricks incorporating waste tire rubber particles were prepared to improve sustainability and optimize the balance between mechanical performance and hydraulic behavior. Orthogonal experiments and response surface methodology were used to investigate the effects of aggregate-to-binder ratio (A/B), water-to-binder ratio (W/B), rubber content, and rubber particle size on compressive strength and permeability coefficient. Results showed that rubber content dominated compressive strength, while A/B ratio had the greatest influence on permeability. Compressive strength decreased continuously with increasing rubber content and A/B ratio, whereas permeability increased with A/B ratio and showed non-monotonic responses to rubber content and particle size. Response surface optimization identified an optimum mixture: A/B = 3.006, W/B = 0.45, rubber content = 0.103, and rubber particle size = 0.525 mm, yielding a compressive strength of 18.97 MPa and a permeability coefficient of 1.82 mm/s. Validation tests showed relative errors of 1.32% for compressive strength and 3.85% for the permeability coefficient, respectively. SEM and CT analyses revealed that the performance of the permeable concrete bricks was governed by the balance among skeleton integrity, interfacial bonding, and pore connectivity. These findings support the valorization of waste tire rubber in sustainable permeable paving materials.

## 1. Introduction

Rapid urbanization has significantly increased the utilization of impervious pavement materials, which reduces rainwater infiltration and aggravates urban flooding, water pollution, and heat island effects [1,2]. In the context of sponge city construction and sustainable urban drainage, permeable pavement materials have become increasingly important in urban infrastructure. Among them, permeable concrete bricks are widely used in roads, sidewalks, and public squares because of their convenient construction, structural stability, and adjustable permeability [3,4]. Meanwhile, the concept of the “zero-waste city” has promoted the recycling of solid wastes in construction materials, providing new opportunities for the development of sustainable permeable paving products [5,6].

Waste tires are a representative bulk solid waste that occupies large landfill space and poses long-term environmental risks [7,8]. Therefore, converting waste tires into rubber particles and incorporating them into permeable concrete bricks is considered a promising approach for waste reutilization and performance regulation. Previous studies have shown that rubber particles can markedly affect the mechanical and hydraulic behavior of permeable concrete materials [9,10,11]. Appropriate rubber incorporation may improve toughness and durability, whereas excessive rubber content generally reduces compressive strength because of the low stiffness of rubber and the weak interfacial bonding between rubber particles and the cementitious matrix [12,13]. Li et al. [14] showed that rubber particle size is a key factor affecting deformation capacity, strength, and permeability, with fine and coarse rubber particles producing different responses through their distinct packing and interfacial characteristics.

In addition, aggregate characteristics and mixture proportions, such as aggregate-to-binder ratio, water-to-binder ratio, and particle size, are key factors governing pore structure, strength development, and permeability performance [15,16,17,18,19]. Zhou et al. [20] showed that increasing rubber powder content reduced permeability, while an appropriate content of 7.5% improved the compressive and flexural strengths of permeable bricks. Qin et al. [21] further indicated that rubber particle size and content jointly controlled the strength–permeability balance, with the optimal strength obtained at a particle size of 0.15–0.30 mm and a rubber content of 3%.

However, most existing studies have mainly examined the effects of only a few individual factors, and the combined effects and interactions among key variables are still not well understood. In particular, for rubber-modified permeable concrete bricks [22,23], the coupled roles of aggregate-to-binder ratio, water-to-binder ratio, rubber content, and rubber particle size in governing both compressive strength and permeability remain unclear. In addition, although orthogonal experimental design is effective for preliminary screening [24], it is less capable of capturing interaction effects and identifying the optimal range when several variables act simultaneously [25,26].

Therefore, this study integrates orthogonal experimental design with response surface methodology to systematically evaluate the effects of aggregate-to-binder ratio, water-to-binder ratio, rubber content, and rubber particle size on the compressive strength and permeability coefficient of rubber-modified permeable concrete bricks. The aims are to identify the dominant governing factors, elucidate the interaction effects among key variables, and determine an optimized mixture proportion that ensures a balanced improvement in mechanical performance and permeability. The results are expected to provide both theoretical support and practical guidance for the sustainable utilization of waste tire rubber in permeable pavement materials.

## 2. Materials and Methods

**Figure 1:**
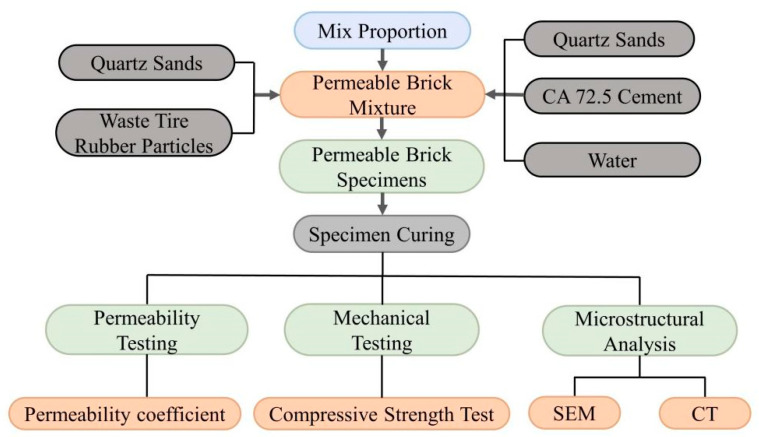
Technical roadmap of this study. Different colors are used only to distinguish different experimental stages.

In this study, quartz sand and waste tire rubber particles were used to prepare permeable concrete bricks with CA 72.5 cement (Weifang Jiuqi Building Materials Co., Ltd., Weifang, China) as the binder. The specimens were cured and then subjected to permeability testing, compressive strength testing, and microstructural analysis. The technical route of this study is shown in Figure 1.

### 2.1. Materials

**Figure 2:**
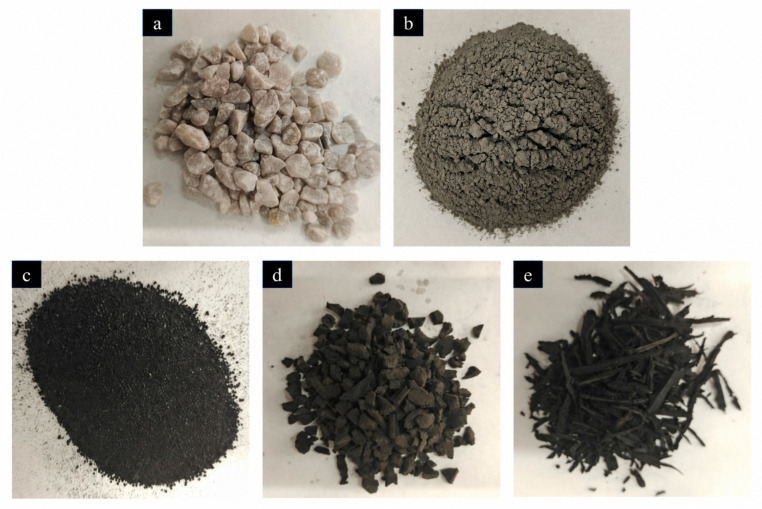
Raw materials used in this study: (**a**) 4–9 mm quartz sand; (**b**) CA 72.5 cement; (**c**) 0.05–1 mm rubber particles; (**d**) 1–4 mm rubber particles; (**e**) 4–9 mm rubber particles.

Quartz sand supplied by Beijing Huabo Station (Beijing, China) was used as the aggregate, as shown in Figure 2a. Its main chemical and physical properties, as provided by the supplier, are summarized in Table 1. The quartz sand had a SiO_2_ content of 99.6% and a mud content of ≤1%, indicating its suitability as the aggregate skeleton in permeable concrete bricks.

Rubber particles were obtained from waste tires through mechanical crushing and sieving, as shown in Figure 2c–e. Three particle size ranges were selected, i.e., 0.05–1 mm, 1–4 mm, and 4–9 mm, to investigate the influence of particle size on the mechanical and permeability properties of permeable bricks.

High-alumina cement (CA 72.5), shown in Figure 2b, was used as the binder. According to the product specifications, its major chemical constituents include Al_2_O_3_, SiO_2_, and Fe_2_O_3_, and it is characterized by rapid hardening and high early strength. The basic physical and mechanical properties of the cement are summarized in Table 2. Tap water was used as the mixing water throughout the experiments.

### 2.2. Experimental Design

#### 2.2.1. Orthogonal Experimental Design

In this study, quartz sand and rubber particles were employed as aggregates, and high-alumina cement was used as the binder. To systematically investigate the influence of mixture parameters, an orthogonal experimental design was adopted with four factors: aggregate-to-binder ratio (A, by mass), rubber-to-binder ratio (B, by mass), rubber particle size (C), and water-to-binder ratio (D, by mass). In this study, rubber content is defined as the mass ratio of rubber particles to cementitious binder (rubber-to-binder ratio, R/B). For example, a rubber-to-binder ratio of 0.10 denotes that the mass of rubber particles is 10% of the mass of CA 72.5 cement. Each factor was assigned three levels, as listed in Table 3, and the experiments were arranged according to an L9(3^4^) orthogonal array [22,25].

The selected ranges of A/B ratio, W/B ratio, rubber-to-binder ratio, and rubber particle size were determined based on preliminary trial mixtures and previously reported mixture proportions for rubber-modified permeable concrete bricks. Trial batches showed that A/B ratios below 3.0 produced excessive paste coating and reduced permeability, whereas values above 3.6 resulted in poor compactness and insufficient bonding. Rubber-to-binder ratios higher than 0.20 led to obvious strength loss and poor molding stability. Therefore, the selected ranges were considered suitable for maintaining both moldability and the strength–permeability balance.

#### 2.2.2. Response Surface Methodology

To further investigate the interaction effects of key factors and optimize the mixture proportion, response surface methodology based on the Box–Behnken design was employed [27]. According to the orthogonal test results, aggregate-to-binder ratio (A), rubber content (B), and rubber particle size (C) were selected as the independent variables, while compressive strength (Y_1_) and permeability coefficient (Y_2_) were taken as the response variables. The experimental matrix was generated using Design-Expert software (version 10; Stat-Ease Inc., Minneapolis, MN, USA), and a second-order polynomial model was established through least-squares regression. ANOVA and response surface analysis were then conducted to evaluate factor significance, quantify interaction effects, and identify the theoretical optimum mixture proportion.

### 2.3. Sample Preparation

**Figure 3:**
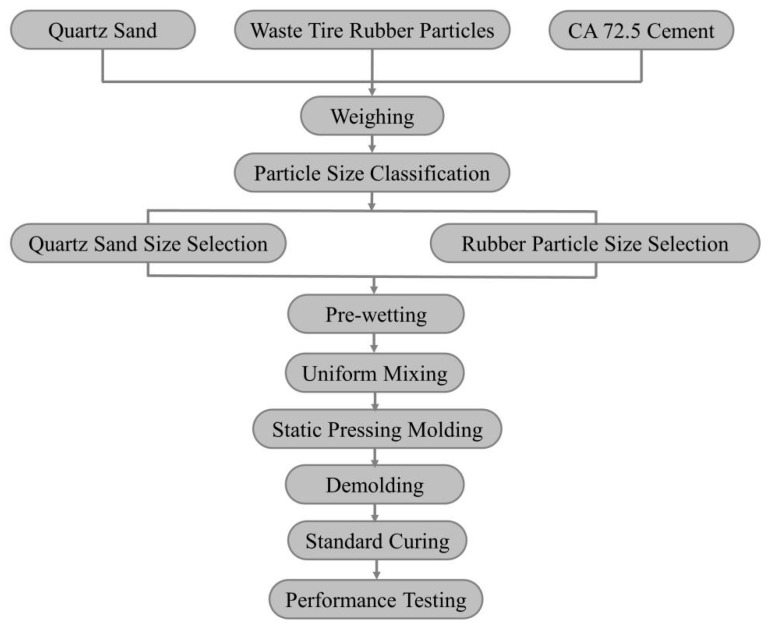
Flow chart of permeable brick manufacturing process.

Based on the selected aggregate-to-binder ratio, water-to-binder ratio, rubber content, and rubber particle size, the required amount of each raw material was calculated and weighed prior to mixing. The dry materials were first blended uniformly (HJW-30, Hebei Xinmingsheng Testing Instrument Co., Ltd., Cangzhou, China), after which the water was added for pre-wetting. Mixing was continued until the materials were uniformly blended. The fresh mixture was placed into 50 mm × 50 mm × 50 mm cubic molds (for compressive strength and porosity tests) and φ100 mm × 50 mm cylindrical molds (for permeability tests), leveled, and compacted using a pressure testing machine (CLY30, Sinotest Equipment Co., Ltd., Changchun, China) by static pressing under 4.5 MPa for 30 s. Three replicate specimens were prepared for each mixture proportion to ensure statistical reliability. After molding, the specimens were demolded, kept at room temperature for 1 d, and subsequently cured at 20 ± 5 °C and 90% relative humidity for 3 d before performance testing [28]. The preparation process of the permeable concrete bricks is shown in Figure 3.

### 2.4. Test Methods

#### 2.4.1. UCS Test

The UCS tests were conducted in accordance with JTG 3441-2024 [28] using an electronic universal testing machine (HLXK Co., Ltd., Jinan, China). Cubic specimens (50 mm × 50 mm × 50 mm, consistent with the molding procedure in Section 2.3) were tested. Prior to loading, a thin layer of petroleum jelly was applied to both specimen ends to reduce friction, and the specimens were carefully positioned between loading plates and aligned centrally. The loading rate was set to 1 mm/min, and load and displacement were recorded continuously until failure. Parallel specimens were tested and the average value was adopted. UCS was calculated using Equation (1):

(1) $$R_{c}=\frac{P_{max}}{A}\times 10$$

where *R*_c_ is the UCS of the sample (MPa), Pmax is the maximum load at failure (kN), *A* is the cross-sectional area of the sample (cm^2^), and 10 is the unit conversion factor.

#### 2.4.2. Permeability Coefficient Test

**Figure 4:**
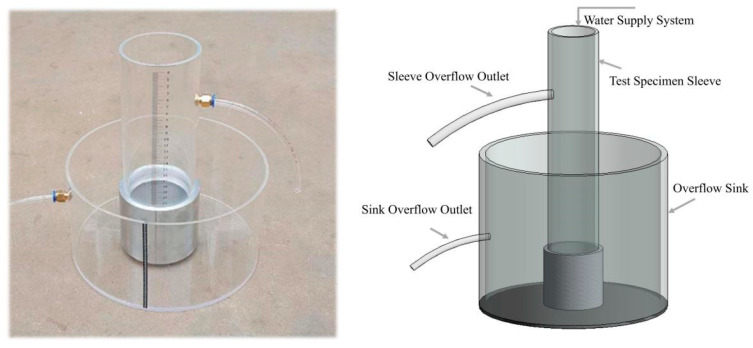
Permeability coefficient test setup.

The permeability coefficient test was conducted in accordance with GB/T 25993-2023 [29] using a permeability tester (GBT25993-A, Cangzhou Huaheng Testing Instrument Co., Ltd., Cangzhou, China). Cylindrical specimens with a diameter of 100 mm and a thickness of 50 mm were used for the constant-head permeability test, consistent with this standard and the molding procedure described in Section 2.3. Prior to testing, the specimens were cured to the specified age and their upper and lower surfaces were cleaned to remove any loose particles or impurities. The specimens were then installed in the testing apparatus, and sealing measures were applied to ensure that water flowed only through the thickness direction of the specimens. The schematic diagram of the permeability coefficient test apparatus is shown in Figure 4.

A constant hydraulic head difference *H* was maintained throughout the test. After the outflow rate became stable, the volume of water *Q* discharged within a time interval *t* was recorded. The permeability coefficient *K_r_* was calculated using Equation (2):

(2) $$K_{r}=\frac{QL}{AHt}$$

where $K_{r}$ is the permeability coefficient (mm/s), Q is the volume of water discharged within time *t* (mm^3^), *L* is the specimen thickness (mm), *A* is the upper surface area of the specimen (mm^2^), *H* is the hydraulic head difference (mm), and *t* is the testing time (s).

#### 2.4.3. SEM Test

The microstructural characteristics of the rubber-modified permeable concrete brick specimens were observed using a scanning electron microscope (S-4700, Hitachi Ltd., Tokyo, Japan) [30]. Representative fragments were taken from the interior of the tested specimens, dried, and coated before observation. The morphology of hydration products, the compactness of the cementitious matrix, and the interfacial bonding between rubber particles and the surrounding paste were analyzed separately.

#### 2.4.4. CT Test

The scanning slice data of permeable concrete brick specimens were obtained using an X-ray computed tomography system (CTNV-5000, Jiangsu Tuochuang Scientific Instrument Co., Ltd., Nanjing, China) [31]. In this study, CT images were mainly used for qualitative observation of the internal pore structure and compactness differences among representative specimens. Due to the limited scope of the present work, quantitative microstructural parameters, such as pore-size distribution, porosity, pore connectivity, and interfacial transition zone characteristics, were not extracted. These quantitative analyses will be considered in future work to further clarify the relationship between pore structure, permeability, and strength development.

## 3. Results and Discussion

### 3.1. Orthogonal Experimental Analysis

#### 3.1.1. Range Analysis

The compressive strength and permeability coefficient of the specimens obtained from the orthogonal experimental design are presented in Table 4.

According to the range analysis results for compressive strength shown in Table 5, the influence of the four factors on the compressive strength of the permeable concrete bricks can be evaluated by comparing the corresponding range values (R). The order of influence was C > A > D > B, with the range values following RC = 2.878 > RA = 2.248 > RD = 1.276 > RB = 1.175. This indicates that rubber particle size was the most influential factor governing compressive strength in the orthogonal experiment, followed by aggregate-to-binder ratio, water-to-binder ratio, and rubber content.

According to the range analysis results for permeability coefficient shown in Table 6, the relative importance of the four factors can also be ranked based on the range values. The order of influence was A > D > C > B, with the corresponding range values *R_A_* = 0.563 > *R_D_* = 0.524 > *R_C_* = 0.488 > *R_B_* = 0.278. This indicates that the aggregate-to-binder ratio had the greatest influence on the permeability coefficient, followed by water-to-binder ratio, rubber particle size, and rubber content.

Based on the range analysis, the optimum combination for compressive strength was an aggregate-to-binder ratio of 3.0, a water-to-binder ratio of 0.45, a rubber content of 0.10, and a rubber particle size of 0.05–1 mm, whereas that for permeability coefficient was an aggregate-to-binder ratio of 3.6, a water-to-binder ratio of 0.40, a rubber content of 0.10, and a rubber particle size of 4–9 mm. Considering both performance indices comprehensively, the final optimized combination was determined as an aggregate-to-binder ratio of 3.6, a water-to-binder ratio of 0.45, a rubber content of 0.10, and a rubber particle size of 4–9 mm, which provided the best overall performance within the investigated range.

#### 3.1.2. Effect of Different Factors on UCS

**Figure 5:**
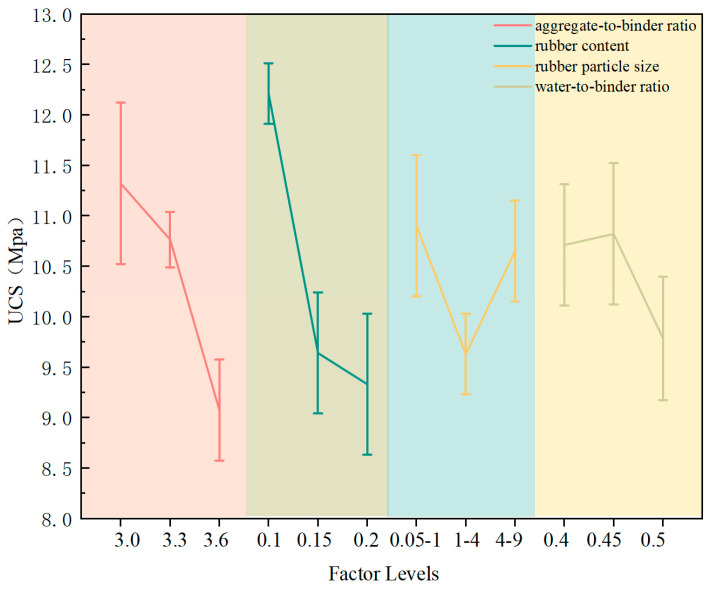
Trends of compressive strength with different factors. Different colors represent different mixture factors. Error bars represent ±1 standard deviation, estimated from the coefficient of variation (C.V. = 5.3%) obtained from the RSM center-point replicates.

The variation associated with rubber particle size did not follow a monotonic trend, but instead showed an initial decrease followed by a subsequent increase (Figure 5). Specifically, the compressive strength decreased from 0.05–1 mm to 1–4 mm and then increased at 4–9 mm, with the minimum value occurring at 1–4 mm. This non-monotonic trend can be explained by the competing influences of particle packing, interfacial area, and stress distribution within the composite matrix. For the fine fraction (0.05–1 mm), the small particle size and high specific surface area allowed a relatively uniform dispersion, and the particles could partially fill the inter-aggregate voids. Although numerous weak rubber–paste interfaces were introduced, the fine particles did not severely disrupt the geometric interlocking of the quartz sand skeleton, thereby preserving moderate load-transfer efficiency. For the medium fraction (1–4 mm), the rubber particles were too large to effectively fill the voids among the 4–9 mm quartz sand aggregates, yet large enough to act as discrete soft inclusions that interrupted the continuity of the load-bearing skeleton. This size regime maximized interfacial defects and local stress concentration, which promoted crack initiation under load and thus produced the lowest compressive strength.

In contrast, for the coarse fraction (4–9 mm), the number of rubber particles decreased markedly at a fixed rubber content, which reduced the total interfacial area and the cumulative weakening effect. Furthermore, the coarse rubber particles were geometrically comparable to the fine aggregate fraction and could be partially enveloped by the surrounding paste and sand, diminishing their detrimental impact on matrix continuity. Consequently, the compressive strength recovered relative to the 1–4 mm level. Overall, the compressive strength development of the permeable concrete bricks reflected the balance among matrix densification, interfacial weakening, and the geometric compatibility of rubber particles within the aggregate skeleton.

#### 3.1.3. Effect of Different Factors on Permeability Coefficient

**Figure 6:**
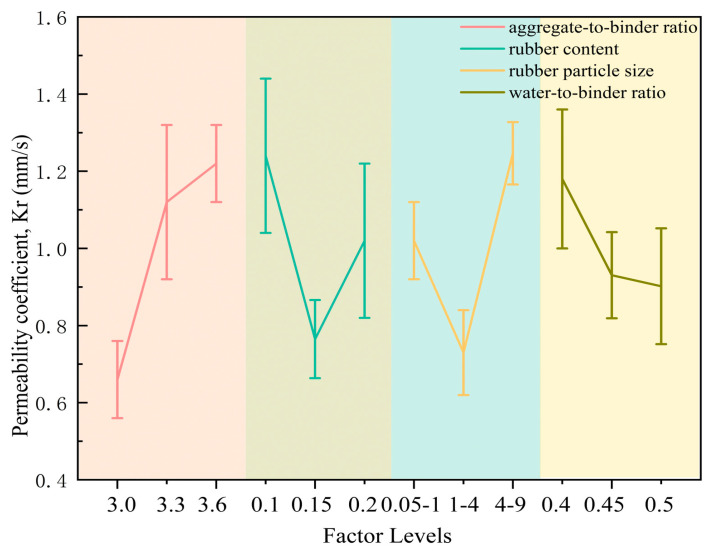
Trends of permeability coefficient with different factors. Different colors represent different mixture factors. Error bars represent ±1 standard deviation, estimated from the coefficient of variation (C.V. = 20.0%) obtained from the RSM center-point replicates.

As shown in Figure 6, the effects of rubber content and rubber particle size both exhibited a typical non-monotonic trend. The effects of rubber content and rubber particle size both exhibited a typical non-monotonic trend. For rubber content, the permeability coefficient first decreased and then increased, with the minimum value observed at a rubber content of 0.15. This indicates that an appropriate amount of rubber particles could partially fill the voids and disrupt pore connectivity, whereas excessive rubber weakened the matrix continuity and increased structural looseness, thereby restoring or even enlarging the effective flow channels. A similar variation was observed for rubber particle size: the permeability coefficient decreased from 0.05–1 mm to 1–4 mm and then increased markedly at 4–9 mm. This behavior can be attributed to the interplay among pore filling, pore channel obstruction, and structural destabilization. Fine rubber particles (0.05–1 mm) possessed a high specific surface area that required more cement paste for coating, leaving less free paste available to fill the inter-aggregate voids. (Figure 6).

Moreover, the abundance of fine particles introduced extensive micro-gaps and weak interfacial transition zones; once interfacial debonding or microcracking occurred, these defects could evolve into preferential flow paths, resulting in a relatively high permeability coefficient. Medium-sized particles (1–4 mm) were most effective in physically obstructing and segmenting the connected pore throats within the quartz sand skeleton without creating excessively large bypass channels. Their dimensions were comparable to the typical pore constrictions in the permeable concrete, enabling them to partially block seepage paths and thereby minimize the permeability coefficient. However, coarse particles (4–9 mm) were comparable in size to the quartz sand aggregates and tended to displace adjacent sand grains during mixing and compaction, inducing a coarser and less stable internal structure with enlarged, well-connected voids. In addition, the poor bonding between the hydrophobic coarse rubber surface and the cement paste generated interfacial gaps that facilitated water flow, leading to a significant increase in permeability.

Overall, the variation in permeability was governed by the combined effects of pore filling by fine particles, pore blocking by medium particles, and pore coarsening induced by coarse particles, reflecting the combined influence of paste coverage, rubber modification, and internal pore connectivity.

### 3.2. Response Surface Analysis

#### 3.2.1. Selection and Design of Factor Levels

Based on the orthogonal results, the aggregate-to-binder ratio (A), rubber content (B), and rubber particle size (C) were selected for response surface analysis, with compressive strength (Y_1_) and permeability coefficient (Y_2_) as the response variables. The orthogonal design identified a preliminary optimum at an aggregate-to-binder ratio of 3.6, a water-to-binder ratio of 0.45, a rubber content of 0.10, and a rubber particle size of 4–9 mm. Accordingly, the water-to-binder ratio was fixed at 0.45, and the remaining three factors were further optimized by response surface methodology. The selected factor levels are listed in Table 7.

#### 3.2.2. Model Development

A Box–Behnken design was employed based on the factor levels listed in Table 7, and the experimental results are presented in Table 8.

**Figure 7:**
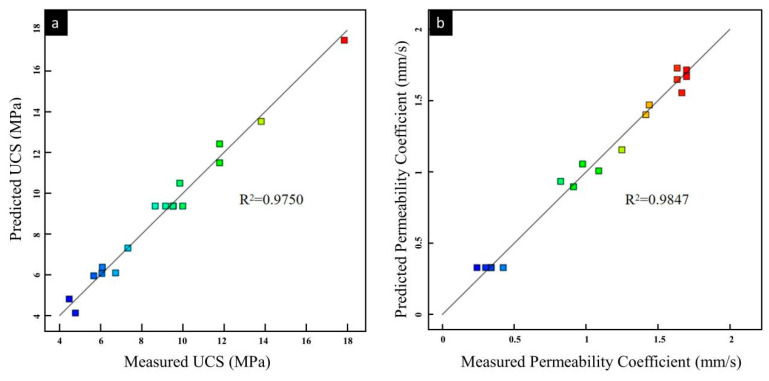
Fitting curves of experimental and predicted values: (**a**) UCS; (**b**) permeability coefficient. The colors of the data points represent the magnitude of the response values, from low values in blue to high values in red. The solid line represents the ideal agreement between measured and predicted values.

Based on the experimental data, quadratic polynomial regression models were established using Design-Expert 10 software through the least-squares method, as given in Equations (3) and (4). The comparison between the measured and predicted values (Figure 7) shows that the data points were closely distributed along the fitted line, confirming the satisfactory agreement between model prediction and experimental observation. The R^2^ values for the 3-day compressive strength and permeability coefficient models were 0.9750 and 0.9847, respectively, demonstrating that both models had high explanatory power and predictive capability within the selected factor range. Therefore, the developed models showed high prediction accuracy and reliability. It should be noted that although the individual replicate raw data for the orthogonal array tests were not tabulated for brevity, the experimental repeatability was quantified using the five center-point replicates in the RSM design, which are listed individually in Table 8 (No. 13–17). The corresponding coefficients of variation were 5.3% for UCS and 20.0% for Kr. Moreover, the high R^2^ (>0.97) and Pred-R^2^ (>0.80) values, together with the insignificant lack-of-fit (*p* > 0.05), collectively confirm the reliability and predictive capability of the established models within the investigated factor ranges [24].

(3) $$Y_{1}\;=\;9.01\;-\;2.86A\;-\;3.48B\;-\;0.3C\;+\;2.23AB\;+\;0.16AC\;+\;1.21BC$$

(4) $$Y_{2}\;=\;0.33\;+0.22\;A\;-0.093B\;+\;0.19C\;+\;0.14AB\;+\;0.14AC\;+\;0.064BC\;+\;0.36A^{2}\;+\;0.69B^{2}\;+\;0.48C^{2}$$

#### 3.2.3. Analysis of Variance

As shown in Table 9, the regression model for 3 d compressive strength was highly significant, with an F-value of 64.90 and a *p*-value lower than 0.0001. Moreover, the lack-of-fit was not significant (*p* = 0.1880), suggesting that the model error was acceptable. The model also exhibited satisfactory goodness of fit, with R^2^, Adj-R^2^, and Pred-R^2^ values of 0.9750, 0.9599, and 0.9060, respectively. In addition, the Adeq Precision value reached 29.004, indicating an adequate signal and good capability for navigating the design space.

As shown in Table 10, the F-value of the regression model for the permeability coefficient is 49.96 with a *p*-value lower than 0.0001 (<0.05), indicating that the quadratic regression model established with permeability coefficient as the response variable is also extremely significant and highly reliable. The lack-of-fit is insignificant. The predicted R^2^ value is reasonably close to the experimental R^2^ value, satisfying the prediction requirement. Moreover, the coefficient of variation is 9.85%, and the signal-to-noise ratio is 17.568, These results indicate that the model possessed acceptable predictive capability.

The statistical indicators for model adequacy are summarized in Table 11.

**Figure 8:**
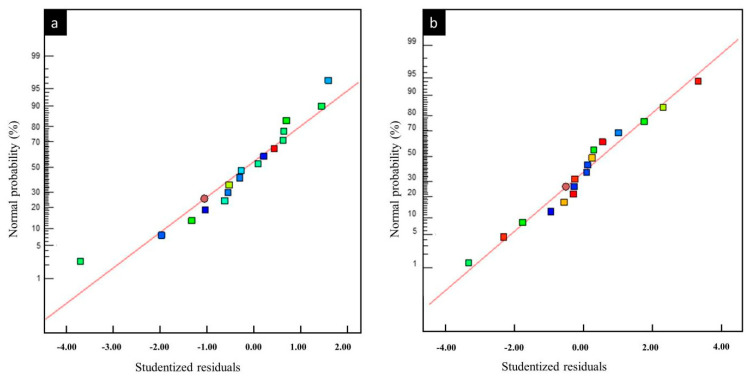
Studentized residual plots of the regression models: (**a**) 3-day compressive strength; (**b**) permeability coefficient. The colors of the data points represent different experimental runs, and the red line represents the normal probability reference line.

The studentized residuals generated by Design-Expert 10 software, defined as the ratio of residuals to their standard deviation, are key diagnostic indicators for evaluating the goodness of model fitting. As shown in Figure 8, for both the 3-day compressive strength and permeability coefficient regression models of the pervious concrete brick specimens, all experimental data points are closely distributed on both sides of the normal probability reference line within a narrow range, without obvious outliers. This indicates that the residual sequences exhibit low variability and conform well to the assumption of normal distribution. The residuals of both models satisfy the normality requirement and their systematic errors are well controlled. Moreover, the fitted curves can accurately capture the nonlinear characteristics of the experimental data, demonstrating that the developed models possess high predictive capability and good engineering applicability.

#### 3.2.4. Interaction Effects of Factors

(1) Interaction effects of factors on compressive strength.

**Figure 9:**
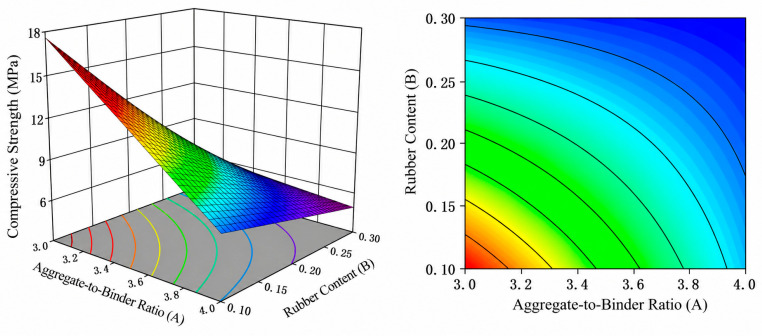
Interaction of aggregate-to-binder ratio and rubber content on UCS. The left panel shows the 3D response surface, and the right panel shows the corresponding contour plot. The color gradient represents the predicted UCS values; red/yellow regions indicate higher UCS, whereas blue/purple regions indicate lower UCS.

Figure 9 presents the contour plot and 3D response surface for the interaction between aggregate-to-binder ratio and rubber content on compressive strength. The relatively smooth contour pattern indicates that the interaction between the two factors was limited, whereas their main effects on compressive strength remained evident. As both aggregate-to-binder ratio and rubber content increased, compressive strength decreased continuously, with a more pronounced decline observed along the rubber-content direction. This synergistic weakening effect can be interpreted through the “dilution–interface” mechanism widely reported in rubberized cementitious systems [12,13,14]. Specifically, a higher aggregate-to-binder ratio reduces the effective paste film thickness coating the aggregate skeleton, which not only weakens the inter-particle bonding but also leaves insufficient mortar to encapsulate the hydrophobic rubber particles [13]. Concurrently, increasing rubber content introduces additional weak interfacial transition zones (ITZs) between the soft rubber phase and the rigid cement matrix; these ITZs act as preferential sites for micro-crack initiation under compressive load, thereby accelerating strength degradation [12,14]. The limited curvature of the contour lines suggests that the two factors operate through partially overlapping pathways—both reduce matrix continuity—rather than through a strong nonlinear coupling [24].

**Figure 10:**
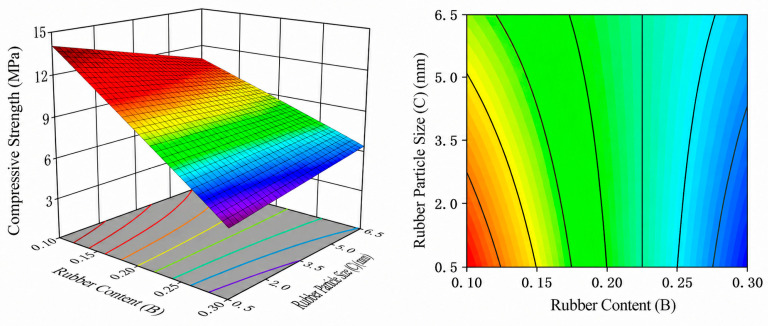
Interaction of rubber content and rubber particle size on UCS. The left panel shows the 3D response surface, and the right panel shows the corresponding contour plot. The color gradient represents the predicted UCS values; red/yellow regions indicate higher UCS, whereas blue/purple regions indicate lower UCS.

Figure 10 presents the contour plot and 3D response surface for the interaction between rubber content and rubber particle size on compressive strength. The curved contour lines indicate that the interaction between the two factors was evident within the investigated range, while the steeper gradient along the rubber-content direction suggests that compressive strength was more sensitive to rubber content than to rubber particle size. The observed interaction aligns with the findings of Li et al. [14], who reported that fine rubber powder (400–600 µm) and coarse rubber particles (10–15 mm) exhibit distinct load-transfer behaviors in pavement base materials. In the present study, at low rubber contents, the detrimental effect of particle size was moderated because the sparse rubber inclusions were surrounded by a relatively intact cementitious matrix, which could still bridge stress across the weak interfaces [22]. However, as rubber content increased, the probability of particle contact and overlap rose sharply; under these conditions, medium-sized particles (1–4 mm) created the most severe geometric incompatibility with the 4–9 mm quartz sand skeleton, generating stress concentrations at sharp contact points and promoting early crack coalescence [20,21]. By contrast, fine particles (0.05–1 mm) could be more uniformly dispersed, whereas coarse particles (4–9 mm) reduced the total interfacial area per unit volume; both scenarios alleviated the stress-amplification effect relative to the medium fraction [14,22]. This size-dependent transition from “dispersed inclusion” to “interconnected weak phase” explains the evident interaction captured by the response surface [23].

(2) Interaction effects of factors on permeability coefficient

**Figure 11:**
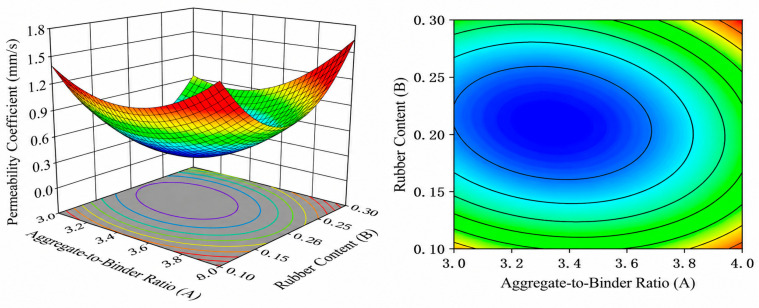
Interaction of aggregate-to-binder ratio and rubber content on permeability coefficient. The left panel shows the 3D response surface, and the right panel shows the corresponding contour plot. The color gradient represents the predicted permeability coefficient values; red/yellow regions indicate higher permeability, whereas blue regions indicate lower permeability.

Figure 11 presents the 3D response surface and contour plot for the interaction between aggregate-to-binder ratio and rubber content on the permeability coefficient. The curved closed contour lines indicate that the interaction between the two factors was evident within the investigated range. The permeability coefficient showed a typical quadratic response, decreasing initially and then increasing with the combined variation of aggregate-to-binder ratio and rubber content, with the minimum value occurring near the intermediate levels of both factors. This nonlinear coupling reflects the competing processes of pore blocking and pore network reconstruction. At a low aggregate-to-binder ratio, the abundant paste tends to fill the inter-aggregate voids; when combined with a moderate rubber content, the rubber particles can partially plug the residual pore throats, thereby reducing hydraulic connectivity [21,25]. However, when both factors shift toward their upper levels, the combined reduction in paste volume and the increased rubber dosage destabilize the aggregate skeleton: the insufficient mortar film cannot maintain particle bonding, while the excessive rubber introduces interfacial gaps and internal looseness that re-establish continuous seepage channels [13,20]. Qin et al. [21] observed a similar trade-off in orthogonal experiments on recycled-aggregate permeable concrete, noting that permeability is governed not merely by total porosity but by the topological connectivity of the pore network—a principle consistent with the quadratic trend observed here.

**Figure 12:**
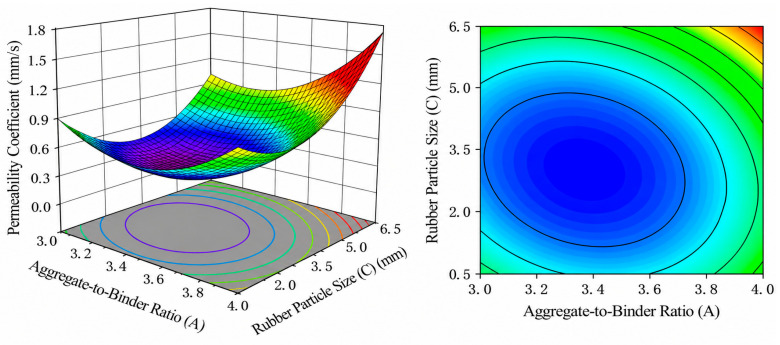
Interaction of aggregate-to-binder ratio and rubber particle size on permeability coefficient. The left panel shows the 3D response surface, and the right panel shows the corresponding contour plot. The color gradient represents the predicted permeability coefficient values; red/yellow regions indicate higher permeability, whereas blue regions indicate lower permeability.

Figure 12 presents the 3D response surface and contour plot for the interaction between aggregate-to-binder ratio and rubber particle size on the permeability coefficient. The nearly circular contour lines indicate that the interaction between the two factors was relatively weak, although their combined effect on permeability was still evident. The permeability coefficient exhibited a quadratic response, decreasing initially and then increasing with the variation of the two factors, with the minimum value located near the intermediate levels. This interaction can be attributed to the partly different action mechanisms of the two variables: the aggregate-to-binder ratio primarily dictates the initial porosity of the skeletal framework [15,16,17,18,19], whereas rubber particle size modulates the local pore geometry through physical obstruction or interfacial debonding [12,20]. Zhou et al. [20] demonstrated that rubber powder content and particle size influence permeable bricks through independent pathways—powder content alters the matrix continuity, while particle size affects the tortuosity of flow channels. In the present study, medium-sized rubber particles (1–4 mm) were most effective in increasing flow path tortuosity at intermediate aggregate-to-binder ratios, because their dimensions matched the characteristic pore constrictions. At high aggregate-to-binder ratios, however, the enlarged initial voids overshadowed the tortuosity effect, causing permeability to rise regardless of particle size [20,25].

**Figure 13:**
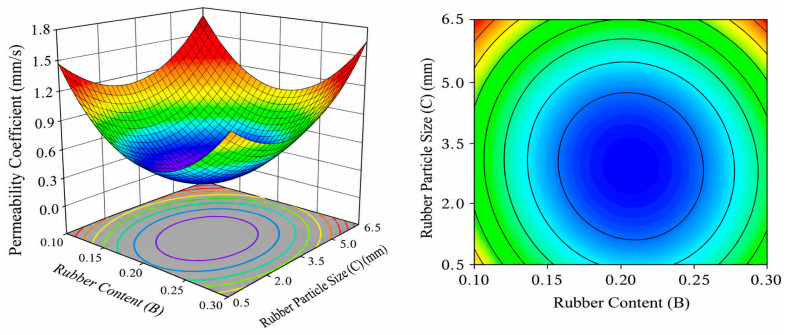
Interaction of rubber content and rubber particle size on permeability coefficient. The left panel shows the 3D response surface, and the right panel shows the corresponding contour plot. The color gradient represents the predicted permeability coefficient values; red/yellow regions indicate higher permeability, whereas blue regions indicate lower permeability.

Figure 13 depicts the interaction between rubber content and rubber particle size on permeability. The nearly circular contour lines and steep response surface suggest a visible but statistically insignificant interaction. As rubber content increases, the influence intensity of particle size on permeability first increases and then decreases. This behavior can be explained by the transition from “pore-clogging” to “interface-channel” dominance as rubber content increases. At low rubber contents, fine particles (0.05–1 mm) tend to distribute within the paste film and partially obstruct narrow pore throats, a mechanism consistent with the pore-filling model proposed for fine rubber powders in cementitious matrices [12,22]. As rubber content rises, the accumulation of hydrophobic coarse particles (4–9 mm) disrupts the aggregate packing arrangement and creates interfacial gaps around the rubber–paste boundaries; these gaps evolve into preferential flow channels because the hydrophobic rubber surface repels the cement paste, preventing effective void sealing [12,13]. The statistically insignificant *p*-value (0.2570) for this interaction (Table 10) suggests that, although the 3D surface exhibits visual curvature, the statistical evidence was insufficient to confirm this interaction, possibly due to the heterogeneity of pore distributions—a limitation also noted in RSM studies of rubberized concrete by Sinkhonde et al. [23] and Mohammed et al. [24]. Nevertheless, the trend supports the hypothesis that the microstructural evolution of rubber-modified permeable bricks is governed by the competition between physical pore filling and chemically driven interfacial debonding [22].

#### 3.2.5. Model Optimization and Validation

Based on the established regression models, the maximum values of compressive strength and permeability coefficient were set as optimization objectives with equal importance. The factor levels were constrained within the ranges of the original experimental design. The numerical optimization module in Design-Expert software was used to solve the model, and the optimal results are summarized in Table 12.

Considering practical engineering conditions, the optimal parameters were adjusted to an aggregate-to-binder ratio of 3.0, a rubber content of 0.10, and a rubber particle size of 0.5 mm. Three parallel experiments were conducted under these conditions, and the results are listed in Table 13. The measured average 3-day compressive strength and permeability coefficient were 18.720 MPa and 1.890 mm/s, respectively. The relative errors between experimental and predicted values were 1.32% and 3.85%, both within 5% [24,28], confirming good agreement between prediction and experimental data and demonstrating the high accuracy and reliability of the proposed optimization model.

To clarify the engineering relevance of the optimized mixture, the obtained properties were further compared with existing standards and typical performance ranges reported for pervious concrete [4,10]. The experimentally validated permeability coefficient of 1.890 mm/s, equivalent to 0.189 cm/s, is considerably higher than the minimum permeability requirement for permeable paving products. For example, GB/T 25993-2023 [29] classifies permeable paving products into permeability grades A and B, with the minimum permeability coefficients of 2.0 × 10^−2^ cm/s and 1.0 × 10^−2^ cm/s, respectively. Therefore, the optimized mixture exhibited a permeability approximately nine times higher than the minimum requirement for Grade A, indicating that sufficient drainage capacity can be maintained even after the incorporation of waste tire rubber particles.

In terms of mechanical performance, the optimized 3-day compressive strength of 18.97 MPa falls within the typical compressive strength range reported for pervious concrete and is close to the C20 level commonly adopted in low-traffic permeable pavement applications. This suggests that the optimized bricks show potential for use in pedestrian pavements, sidewalks, public squares, parking areas, and sponge-city drainage facilities rather than heavy-duty traffic pavements. It should be noted that GB/T 25993-2023 evaluates the strength grade of permeable paving bricks mainly through splitting tensile strength or flexural strength, whereas this study focused on 3-day compressive strength. Therefore, the present comparison is intended to demonstrate engineering relevance rather than to claim full standard certification. Future work should include 28-day compressive strength, flexural strength, splitting tensile strength, abrasion resistance, freeze–thaw durability, and field drainage performance to further verify practical applicability.

### 3.3. Microstructure of Permeable Concrete Bricks

Microstructural analysis was carried out mainly from the perspectives of rubber content and rubber particle size, since these two factors played dominant roles in governing the mechanical and permeability properties of the permeable concrete bricks.

**Figure 14:**
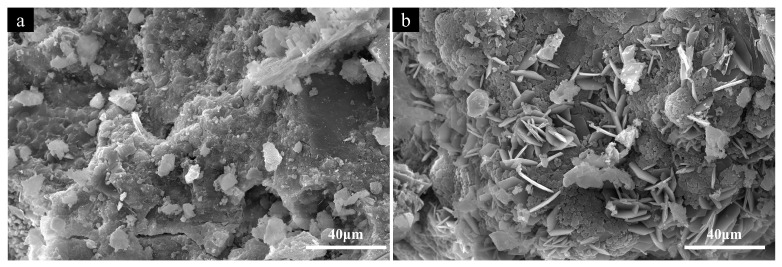
Microstructural characterization of specimens with varied rubber content and particle size. SEM images of specimens with (**a**) high rubber content, small particle size (3000× magnification); (**b**) low rubber content, large particle size (3000× magnification).

To clarify the microstructural mechanism of the rubber-modified permeable concrete bricks, representative specimens with different rubber contents and particle sizes were selected for SEM and CT analyses. The SEM results, as shown in Figure 14, showed that the specimens with lower rubber content and relatively larger particle size exhibited a denser cementitious matrix and a more continuous aggregate–paste skeleton, whereas the specimens with higher rubber content and smaller particle size showed more interfacial defects, microcracks, and a looser internal structure. These observations corroborate the macroscopic trends identified in Section 3.1 and Section 3.2. The denser microstructure in the low-rubber, large-particle specimens (Figure 14b) explains their superior compressive strength (Table 4, Tests 1 and 8): the reduced total interfacial area minimized the cumulative weakening effect of the hydrophobic rubber–cement boundaries, allowing the high-alumina paste to form effective bonding bridges between quartz sand grains [12,13,22].

Conversely, the abundance of fine rubber particles in the high-content, small-size system (Figure 14a) multiplied the weak interfacial transition zones (ITZs), which not only reduced load-transfer efficiency but also served as nucleation sites for microcracks under mechanical loading [7,12]. This interpretation is consistent with the findings of Zhang et al. [7], who identified poor rubber–matrix adhesion as the primary factor limiting the compressive strength of rubberized concrete.

**Figure 15:**
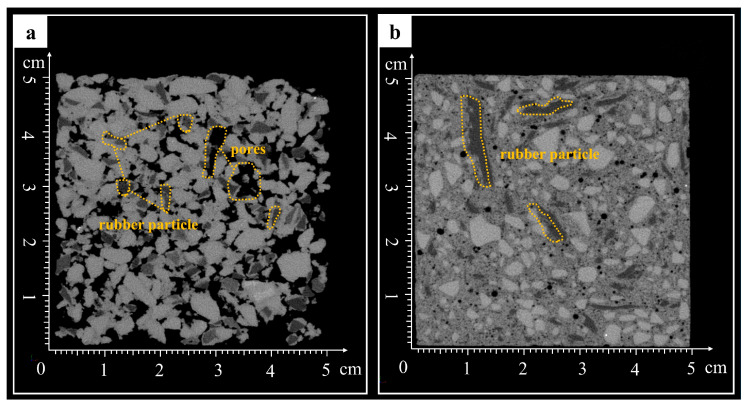
CT scan images of specimens with (**a**) high rubber content, small particle size; (**b**) low rubber content, large particle size.

The CT results, as shown in Figure 15, further confirmed the differences in internal pore structure. Specimens with lower rubber content and larger particle size (Figure 15b) displayed a relatively denser internal structure with isolated, low-tortuosity pores, which is conducive to moderate permeability without excessive strength loss. In contrast, the high-rubber, small-particle specimens (Figure 15a) exhibited a more developed connected pore network with higher topological connectivity—a microstructural signature that directly accounts for the non-monotonic permeability trends observed in Section 3.1.3. At high fine-rubber contents, the inability of the paste to fully coat the numerous hydrophobic surfaces left interfacial gaps that coalesced into continuous seepage channels, thereby increasing permeability despite the physical presence of pore-filling particles. This “interface-channel” mechanism has been previously hypothesized by Qiao et al. [22] in their study on microstructure-engineered porous cementitious composites with recycled tire particles, and the present CT evidence provides visual evidence supporting this mechanism. Taken together, the SEM and CT analyses reveal that the macroscopic performance of the permeable concrete bricks is governed by the balance between skeleton integrity and pore connectivity, with rubber content and particle size acting as the dominant microstructural regulators through their control of interfacial area and pore topology [22,31].

## 4. Conclusions

In this study, response surface methodology was employed to optimize the mixture proportions of permeable concrete bricks, considering both macroscopic performance and microstructural characteristics. Based on the experimental results, the following conclusions can be drawn:

(1) The optimal mixture proportions were identified as an aggregate-to-binder ratio of 3.006, a water-to-binder ratio of 0.45, a rubber content of 0.103, and a rubber particle size of 0.525 mm. Under these conditions, the compressive strength and permeability coefficient reached 18.97 MPa and 1.82 mm/s, respectively.
(2) The compressive strength decreased with increasing aggregate-to-binder ratio and exhibited a non-monotonic trend with water-to-binder ratio, increasing initially and then decreasing. Although the incorporation of rubber particles reduced mechanical strength, appropriate control of particle size effectively mitigated this negative effect.
(3) The permeability coefficient increased with the aggregate-to-binder ratio. Rubber content exhibited a dual effect: moderate amounts contributed to partial pore filling, while excessive rubber content or larger particle sizes increased pore connectivity and permeability. A particle size of approximately 0.5 mm provided a favorable balance between strength and permeability.
(4) Microstructural analysis revealed that the mechanical performance was primarily governed by the integrity of the load-bearing skeleton and interparticle interlocking. Due to their low stiffness and hydrophobic nature, rubber particles weakened the interfacial bonding with the cement matrix, leading to the formation of weak interfacial transition zones. This resulted in reduced structural continuity, decreased load-transfer capacity, and increased permeability.
(5) The utilization of waste tire rubber offers significant environmental benefits by reducing landfill burdens and promoting a circular economy. The optimized bricks show potential for application in low-traffic pavements, parking lots, and sustainable drainage systems, contributing to sponge city development through enhanced stormwater infiltration and urban heat island mitigation.
(6) Future research should investigate long-term durability under freeze–thaw cycles, chemical erosion, and sustained mechanical loading, alongside surface modification techniques to improve rubber-matrix interfacial bonding. Additionally, life-cycle assessment, economic feasibility, and the incorporation of supplementary cementitious materials warrant further study to enhance sustainability and field performance [32].

## Figures and Tables

**Table 1:** Chemical and physical properties of quartz sand.

Property	Refractoriness (°C)	Acid Resistance (μg/L)	SiO_2_ Content (%)	Mud Content (%)	Uniformity Coefficient
Value	1700 °C	20 μg/L	99.6	≤1	≤1.8

**Table 2:** Physical and mechanical properties of CA 72.5 cement.

Property	Specific Surface Area (m^2^/kg)	Setting Time (min)		Flexural Strength (MPa)		Compressive Strength (MPa)		Soundness
Value	421	Initial setting time	Final setting time	3 d	28 d	3 d	28 d	Qualified
		105	230	7.7	8.7	63.2	72.5	

**Table 3:** Orthogonal test factors level table.

Levels	A	B	C	D
1	3	0.10	0.05–1	0.4
2	3.3	0.15	1–4	0.45
3	3.6	0.20	4–9	0.5

**Table 4:** Orthogonal experiment results.

Test No.	A	B	C	D	UCS (MPa)	*K_r_* (mm/s)
1	3	0.1	0.05–1	0.4	13.964	1.089
2	3	0.2	1–4	0.45	9.928	0.333
3	3	0.15	4–9	0.5	10.074	0.561
4	3.3	0.2	4–9	0.4	10.296	1.572
5	3.3	0.15	0.05–1	0.45	10.963	0.803
6	3.3	0.1	1–4	0.5	11.093	0.984
7	3.6	0.15	1–4	0.4	7.878	0.877
8	3.6	0.1	4–9	0.45	11.572	1.633
9	3.6	0.2	0.05–1	0.5	7.772	1.161

Note: *K_r_* represents the permeability coefficient.

**Table 5:** Range analysis of compressive strength.

Level Number	A	B	C	D
$K_{1}$	33.967	32.138	36.629	32.699
$K_{2}$	32.352	32.464	28.916	28.899
$K_{3}$	27.222	28.939	27.996	31.942
$k_{1}$	11.322	10.713	12.210	10.900
$k_{2}$	10.784	10.821	9.639	9.633
$k_{3}$	9.074	9.646	9.332	10.647
*R*	2.248	1.175	2.878	1.267
Order of factors	C > A > D > B			

Note: K_i_ represents the sum of the test results for a given factor at level i, k_i_ represents the corresponding mean value, and R is the range, calculated as the difference between the maximum and minimum k values for each factor.

**Table 6:** Range analysis of permeability coefficient.

Level Number	A	B	C	D
$K_{1}$	1.983	3.539	3.706	3.054
$K_{2}$	3.359	2.769	2.241	2.194
$K_{3}$	3.671	2.706	3.066	3.766
$k_{1}$	0.661	1.180	1.235	1.018
$k_{2}$	1.120	0.923	0.747	0.731
$k_{3}$	1.224	0.902	1.022	1.255
*R*	0.563	0.278	0.488	0.524
Order of factors	A > D > C > B			

Note: The symbols are expressed the same as in Table 5.

**Table 7:** Factors and levels of the RSM.

Factors	Value		
	−1	0	1
A	3.0	3.5	4.0
B	0.1	0.2	0.3
C	0.5	3.5	6.5

**Table 8:** Experimental design and results of the RSM.

No.	A	B	C (mm)	UCS (MPa)	*K_r_* (mm/s)
1	3	0.1	3.5	17.852	1.415
2	4	0.1	3.5	7.330	1.665
3	3	0.3	3.5	6.066	0.824
4	4	0.3	3.5	4.478	1.633
5	3	0.2	0.5	11.789	0.913
6	4	0.2	0.5	6.089	0.976
7	3	0.2	6.5	11.792	1.088
8	4	0.2	6.5	6.736	1.698
9	3.5	0.1	0.5	13.812	1.439
10	3.5	0.3	0.5	4.780	1.249
11	3.5	0.1	6.5	9.857	1.633
12	3.5	0.3	6.5	5.674	1.698
13	3.5	0.2	3.5	9.540	0.342
14	3.5	0.2	3.5	9.166	0.425
15	3.5	0.2	3.5	8.661	0.340
16	3.5	0.2	3.5	10.000	0.243
17	3.5	0.2	3.5	9.526	0.303

**Table 9:** ANOVA for the regression model of 3-day compressive strength.

Source	SS	Df	MS	F-Value	*p*-Value	Significance
Model	188.99	6	31.50	64.90	<0.0001	***
A-Aggregate-to-binder ratio	65.36	1	65.36	134.67	<0.0001	***
B-Rubber-to-binder-ratio	96.97	1	96.97	199.81	<0.0001	***
C-Rubber particle size	0.73	1	0.73	1.50	0.2491	ns
AB	19.96	1	19.96	41.12	<0.0001	***
AC	0.10	1	0.10	0.21	0.6536	ns
BC	5.87	1	5.87	12.11	0.0059	**
Residual	4.85	10	0.49			
Lack of fit	3.86	6	0.64	2.59	0.1880	
Total	193.84	16				

Note: SS represents the sum of squares, Df represents the degrees of freedom, and MS represents the mean square. ** *p* < 0.01, *** *p* < 0.001; ns, not significant.

**Table 10:** ANOVA for the regression model of 3-day permeability coefficient.

Source	*SS*	Df	MS	F-Value	*p*-Value	Significance
Model	4.83	9	0.54	49.96	<0.0001	***
A	0.37	1	0.37	34.86	0.0006	**
B	0.070	1	0.070	6.51	0.0380	*
C	0.30	1	0.30	27.64	0.0012	**
AB	0.078	1	0.078	7.27	0.0308	*
AC	0.075	1	0.075	6.95	0.0336	*
BC	0.016	1	0.016	1.52	0.2570	
A^2^	0.54	1	0.54	50.53	0.0002	**
B^2^	2.03	1	2.03	189.24	<0.0001	***
C^2^	0.97	1	0.97	90.10	<0.0001	***
Residual	0.075	7	0.011			
Lack of fit	0.058	3	0.019	4.38	0.0937	
Total	4.90	16				

Note: SS represents the sum of squares, Df represents the degrees of freedom, and MS represents the mean square. * *p* < 0.05, ** *p* < 0.01, *** *p* < 0.001; ns, not significant.

**Table 11:** Statistical indicators for model adequacy.

Model	Std. Dev	Mean	R2	Adj-R2	Pred R2	C.V. (%)	Adeq Precision
$Y_{1}$	0.70	9.01	0.9750	0.9599	0.9060	7.73	29.004
$Y_{2}$	0.10	1.05	0.9847	0.9650	0.8064	9.85	17.568

**Table 12:** Optimization results obtained from response surface methodology.

Water-to-Binder Ratio	Aggregate-to-Binder Ratio	Rubber Content (%)	Rubber Particle Size (mm)	UCS (MPa)	K (mm/s)	Desirability
0.45	3.006	0.103	0.525	18.971	1.820	0.96

**Table 13:** Experimental validation results of the optimized mixture proportion.

Variable	3d UCS (MPa)	*K_r_* (mm/s)
Predicted value	18.971	1.820
Experimental value	18.720	1.890
Relative Error	1.32%	3.85%

Note: The validation tests were performed using three parallel specimens, and the relative errors were calculated from the predicted and experimental mean values.

## Data Availability

The data presented in this study are available in the article. Additional raw data are available from the corresponding author upon reasonable request.
